# Development of a transient expression assay for detecting environmental oestrogens in zebrafish and medaka embryos

**DOI:** 10.1186/1472-6750-12-32

**Published:** 2012-06-24

**Authors:** Okhyun Lee, Charles R Tyler, Tetsuhiro Kudoh

**Affiliations:** 1 Biosciences, College of Life and Environmental Sciences, University of Exeter, Exeter, Devon, EX4 4PS, UK

**Keywords:** Oestrogenic endocrine disrupting chemicals, Oestrogen response elements, UAS-GAL4, Transient expression assay, Zebrafish

## Abstract

**Background:**

Oestrogenic contaminants are widespread in the aquatic environment and have been shown to induce adverse effects in both wildlife (most notably in fish) and humans, raising international concern. Available detecting and testing systems are limited in their capacity to elucidate oestrogen signalling pathways and physiological impacts. Here we developed a transient expression assay to investigate the effects of oestrogenic chemicals in fish early life stages and to identify target organs for oestrogenic effects. To enhance the response sensitivity to oestrogen, we adopted the use of multiple tandem oestrogen responsive elements (EREc38) in a *Tol2* transposon mediated Gal4ff-UAS system. The plasmid constructed (pTol2_ERE-TATA-Gal4ff), contains three copies of oestrogen response elements (3ERE) that on exposure to oestrogen induces expression of Gal4ff which this in turn binds Gal4-responsive Upstream Activated Sequence (UAS) elements, driving the expression of a second reporter gene, EGFP (Enhanced Green Fluorescent Protein).

**Results:**

The response of our construct to oestrogen exposure in zebrafish embryos was examined using a transient expression assay. The two plasmids were injected into 1–2 cell staged zebrafish embryos, and the embryos were exposed to various oestrogens including the natural steroid oestrogen 17ß-oestradiol (E_2_), the synthetic oestrogen 17α- ethinyloestradiol (EE_2_), and the relatively weak environmental oestrogen nonylphenol (NP), and GFP expression was examined in the subsequent embryos using fluorescent microscopy. There was no GFP expression detected in unexposed embryos, but specific and mosaic expression of GFP was detected in the liver, heart, somite muscle and some other tissue cells for exposures to steroid oestrogen treatments (EE_2_; 10 ng/L, E_2_; 100 ng/L, after 72 h exposures). For the NP exposures, GFP expression was observed at 10 μg NP/L after 72 h (100 μg NP/L was toxic to the fish). We also demonstrate that our construct works in medaka, another model fish test species, suggesting the transient assay is applicable for testing oestrogenic chemicals in fish generally.

**Conclusion:**

Our results indicate that the transient expression assay system can be used as a rapid integrated testing system for environmental oestrogens and to detect the oestrogenic target sites in developing fish embryos.

## Background

Decades of research have shown that a number of natural and man-made chemicals interfere with the endocrine system and can result in adverse health effects in humans, mammals and fish [1-3]. Wildlife living in, or closely associated with the aquatic environment have been shown to be especially impacted by these so-called endocrine-disrupting chemicals (EDCs), because our freshwaters, estuaries and oceans act as sinks for chemical discharges [4]. Oestrogenic EDCs in the environment are of particular concern. Endogenous oestrogens are a group of closely related steroid hormones essential in the development and functioning of the reproductive system. Extensive work has been conducted on natural (e.g. 17β-oestradiol) and synthetic oestrogens (e.g. 17α-ethinyloestradiol from the contraceptive pill) and their interactions in the vertebrate body, including their tissue distributions, mechanisms of action and pathways of elimination [5-7]. Furthermore, adverse effects of exposure to environmental oestrogens have received considerable research attention in a number of animal species, but this work is largely restricted to adults and juveniles [6,8], and there has been little study of effects on embryos. Nothing is known regarding the relative sensitivities of the different cell types and tissues to oestrogenic EDCs, or other contaminants, during embryogenesis. Furthermore, in spite of the widespread concern for EDCs in the aquatic environment, there are very few bioassay systems that are sufficiently sensitive for accurate prediction of adverse biological effects. Moreover, conventional methods of EDCs detection such as tissue culture [9] and *in vitro* techniques [10,11] are limited in their capacity to elucidate oestrogen signalling pathways and tissue specific physiological impacts.

Teleost fish have three oestrogen receptors (ER), ERalpha, ERbeta-1 and ERbeta-2, that show tissue specific patterns of expression and function in adults [12-14]. The different ER subtypes are also widely expressed in body tissues in early life stages, from embryos to young larvae [15], suggesting crucial roles of these signalling pathways in early development. Indeed, recently it was found that knockdown of ER-beta2 in the zebrafish suppressed normal development of the lateral line neuromast cells [16]. Endogenous oestrogen receptors activated by oestrogenic chemicals bind to oestrogen response elements within regulatory regions of oestrogen-responsive genes. Response elements are recognized by nuclear transcription factors, including members of the steroid/nuclear receptor super family that then, together with various other regulatory factors mediate transcription of the associated downstream genes [17].

The adopted model in this work, the zebrafish (*Danio rerio*) has become one of the most commonly used animals for examining effects of aquatic pollutants [18]. Furthermore, with the available genomic resources and suitability of this species for molecular manipulations, the zebrafish has been applied more widely for research in developmental biology and understanding of disease processes. The medaka (*Oryzias latipes*) is another model species widely used in ecotoxicology research and for the development of transgenic techniques. Many studies have shown that early life stages of fish have the greatest sensitivity to environmental contaminants and effects on development in the zebrafish and medaka are greatly facilitated by the fact that their embryos are transparent. [19-21].

Use of tissue-specific promoters has become a powerful tool for studies on endogenous gene expression [22] and to analyse the function of promoters [9,23-25] and transgenic fish have become an established technique in developmental analyses. This generally includes using a specific promoter and a green fluorescent protein (GFP) or a luciferase reporter gene. To improve the efficiency of the sensitivity, tissue specificity and ease of generating transgenic fish, various manipulated gene systems have recently been introduced. One of these is the Gal4-UAS system. This is now used widely for the over expression of transgenes in various transgenic animals, including zebrafish [26]. This system comprises a two-part expression system that utilises the yeast transcription activator protein Gal4 and its target sequence UAS (Upstream Activated Sequence), to which Gal4 binds to activate gene transcription [27,28]. The *Tol2* transposon system, originally identified in the medaka [29], has recently been used to enhance the success rate of DNA integration into the zebrafish genome [30,31]. This is illustrated in the work of [30,32]) where used of *Tol2* increased the transgenesis rate of linear DNA from 5% to 50%.

Transgenic zebrafish have considerable potential for use in aquatic ecotoxicology to screen and test for hormone mimics and potentially to develop a more advanced system for assessing health impacts of chemicals. As a consequence there has been considerable activity in a number of laboratories to develop transgenic zebrafish as tools for screening and testing of chemicals [33-36]. Transgenic fish have the advantage that tissue specific effects of EDCs can be identified to allow for more directed and detailed studies to inform on health outcomes. However, it is time consuming, both to produce and maintain the stable transgenic lines. As a consequence, a number of studies have investigated the use of transient expression assays to examine the spatial and temporal expression of reporter genes that are fused to the regulatory regions of various genes in zebrafish embryos [37,38]. To date, biosensor transgenic fish (TG fish) have only been generated in the zebrafish and medaka, and such technology has not been applied widely to other fish species. In theory, however, having developed the technology for these model species, it would be possible to develop a functional ‘transient assay’, by which vector DNA is transiently introduced into the fish embryo, in almost any fish species where the eggs produced can physically be injected, and thus examine the effect of chemical exposure specifically in those fish species.

To date, there have not been any reported transient expression assays for the detection of oestrogenic EDCs. The transient expression assay process normally involves the injection of fertilised embryos with a construct and followed by assaying of the response (e.g. GFP or Luciferase) once the embryos/larvae have reached the desired stage of development. A major advantage of the transient assay would be that the technique is applicable to a variety of fish species for examining tissue, stage and chemicals specific responses without the need to generate TG fish lines.

With a plan to develop a rapid and sensitive transient expression assay system to evaluate the oestrogenic activity of environmental chemicals in different fish embryos, we utilised a Gal4ff-UAS system that incorporates a two-step signal amplification process and a synthetic oestrogen responsive element with 3EREs. A major aim in this initial work was to make a construct that was highly responsive to oestrogenic EDCs. We then examined the responsiveness of the oestrogen response elements and tissue and stage specific responses in zebrafish using a transient expression assay. Finally, we tested our plasmid in another fish species, the medaka, to show that transient expression assay is suitable for observation of effects of oestrogenic compounds in other fish test species.

## Results and discussion

### Construction of reporter gene vectors with oestrogen responsive promoters

In the sequence of results reported, we first developed a novel transient assay system using a synthetic oestrogen responsive element, *Tol2* and Gal4-UAS systems and the GFP reporter gene, responsive to environmental oestrogen. We then investigated the functional capability of this construct and response to a range of potent and weak environmental oestrogens using a transient expression assay using green fluorescent microscopy in zebrafish embryos/larvae. Finally, we proofed our vector system further in another fish species, the medaka.

**Figure 1:**
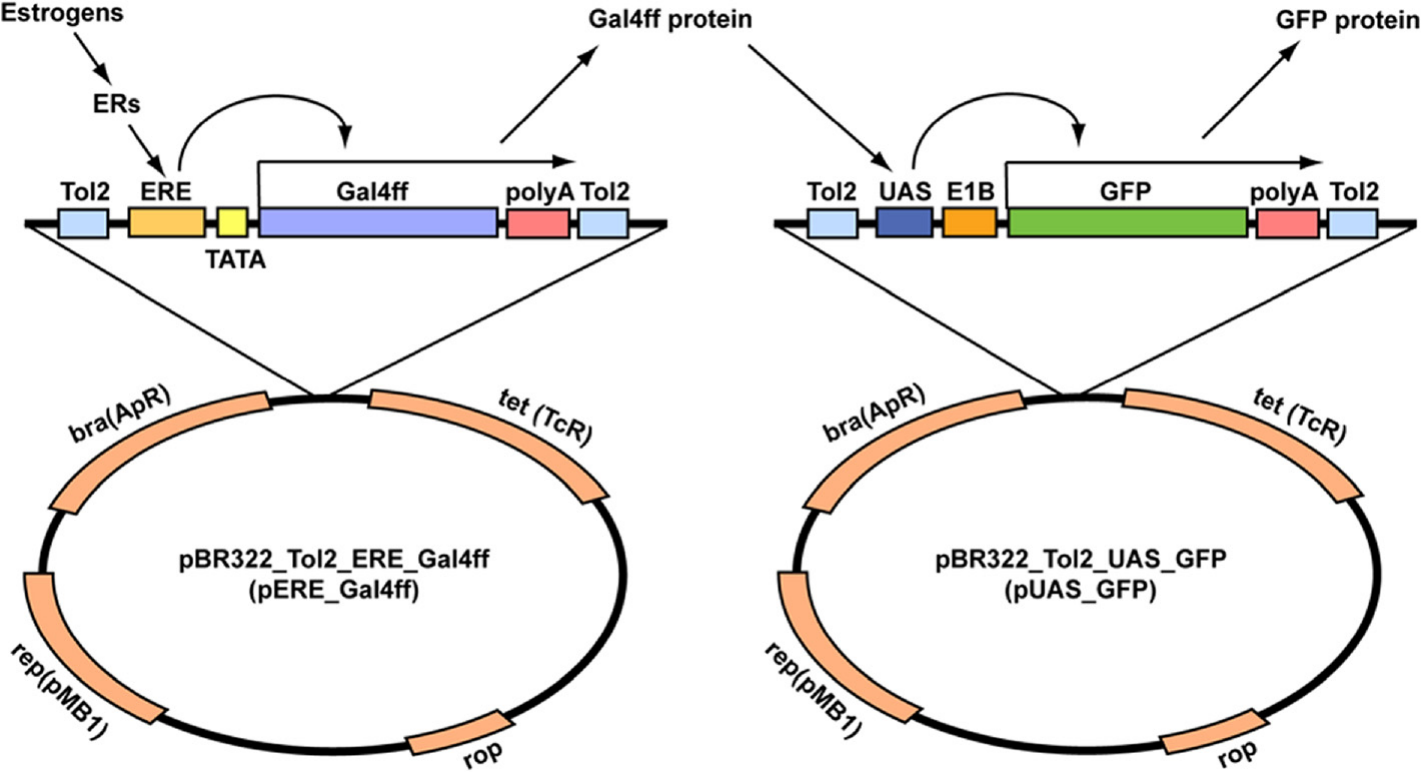
**Construction of reporter gene vectors with an oestrogen responsive promoter.** 3ERE, TATA and Gal4ff were inserted into the *BamHI* and *NotI* sites of the plasmid pBR322 vector containing a *Tol2* element. Xeno-oestrogens bind to and activate the ER. The homodimer complex binds to specific DNA sequences called the oestrogen response element (ERE) within regulatory regions of oestrogen responsive genes. Transcription is induced and messenger RNA is translated into protein. GAL4ff activates “UAS (Upstream Activated Sequence) – GFP (Green Fluorescent Protein), in this “two-step” activation process (Step 1: Oestrogen induces synthesis of GAL4ff protein, step 2: GAL4ff protein induces synthesis of GFP).

The construct produced for detecting oestrogen is shown in Figure 1. Specific primers of EREc38 were run in PCR reactions to produce the EREs. The EREs were 38 base pair (bp) sequences that consisted of a 17-bp inverted repeat 5’-CAGGTCA nnn TGACCTG-3’ followed by an AT-rich sequence [39]. Other studies that have made TG fish for screening of oestrogen chemicals have used various (and different) promoters, including for the following genes, *vitellogenin* (VTG) [34], *choriogenin L* (ChgL) [40] and aromatase B [41]. Legler et al. (2000) [42] developed a transgenic zebrafish line that expressed the reporter gene luciferase (LUC) for examining oestrogenic compounds. Although the construct they used, pEREtata-LUC, was sensitive, luciferase is not as stable as GFP. The luciferase half-life varies from 3 to 14 h [43], as it strongly depends on the host cell and the gene construct. Furthermore, measurement of luciferase is a bioassay that requires tissue homogenisation. In contrast, GFP can be detected in living cells/organisms. The use of GFP in intact fish also has advantages over use of *in vitro* transfected cell cultures, because it allows for integration of toxicodynamic and toxicokinetic factors into the response [44]. The constructs that have been used in previous studies for developing oestrogen response systems, such as pEGFP-ChgL [45], pZVTG1-EGFP [34] are also liver specific genes.

In a mammalian cell culture system, Sathya et al. (1997) [39] showed that a construct with 3EREs produced an enhanced expression compared with 2EREs and 4EREs, especially in response to the natural oestrogen, oestrdaiol-17β (E_2_). We therefore similarly adopted three copies of ERE in our construct. For the transcriptional initiation, the TATA box sequence was inserted at 158 bp upstream of the Gal4ff start codon and the *Tol2* transposon system was adopted to provide a more efficient transient reporter analyses. After the sequential synthesis of five successive constructs, we generated a novel plasmid vector system, p3ERE-TATA-Gal4ff containing, *Tol2* and a poly A tail and employing two steps for amplification of the signal (Figure 1) where Gal4ff activates “UAS-GFP’. In the activation process, the first step, binding of oestrogens to ER and subsequent binding to ERE, induces the synthesis of GAL4ff protein and in the second step Gal4ff protein induces synthesis of GFP. This two-step process allows amplification of the resulting signal which, in theory, should give rise to a more “sensitive” transient expression assay system for detecting oestrogen chemicals than has been developed previously. In our approach, we used Gal4ff rather than the more commonly used Gal-VP16/UAS system, because GAL4ff is reported to be less toxic than GAL4-VP16 in zebrafish cells [46].

### Induction of GFP in the zebrafish embryo transient expression assay

In the transient microinjection studies, expression of gene in zebrafish and medaka embryos was mosaic because the microinjected recombinant DNAs were not integrated to all cells. We minimised this problem by flanking the transgene with a *Tol2* transposon and we were then able to identify target tissues for oestrogens in early life stages more effectively [31].

No differences in mortality rate were observed between both control and oestrogen chemical treated groups (> 95% survival), with the exception for 100 μg NP/L where the most embryos died by 24 hpf. The lack of toxicity effects in zebrafish embryos at the exposure concentrations adopted (with the exception of 100 ug NP/L) compared very favourably with previously reported findings (e.g. Scholz and Gutzeit (2000) for EE_2_[47] and Kishida et al. (2001) for E_2_[48]).

GFP expression was seen at 24 h after exposure to EE_2_ (data not shown), albeit at low level, whereas there was no GFP expression detected in the controls at this time. There was stronger and more specific tissue GFP expression in EE_2_ treated embryos at 72 and 96 hpf, focused mainly in the liver, muscle and heart and this was therefore adopted as the time period for assessing tissue specific effects of oestrogens in the transient assay.

**Figure 2:**
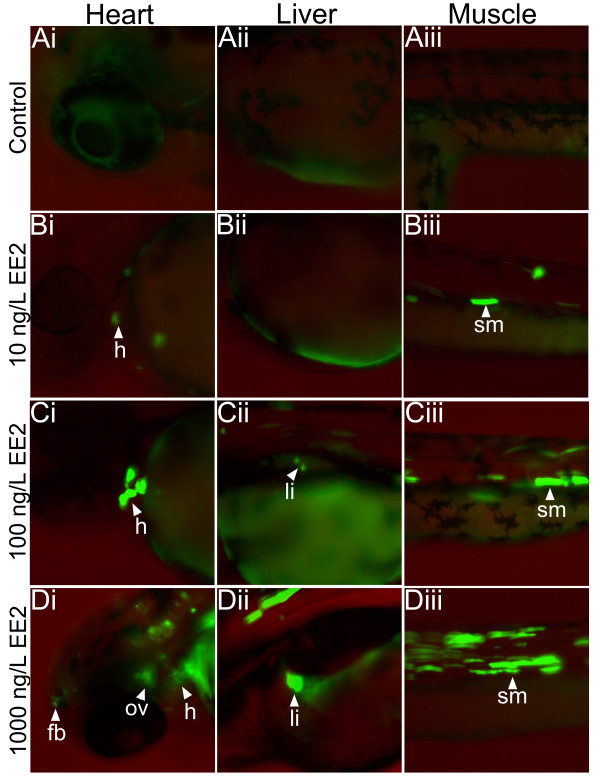
**Expression of GFP in zebrafish embryos in the transient expression assay using the mixture of the plasmid pERE-Gal4ff containing the*Tol2*transposase mRNA and the plasmid pUAS-GFP.** Fertilised zebrafish eggs were microinjected at the 1–2 cell stages with the mixture of two plasmids. Injected embryos were then exposed for up to 72 hour post-fertilisation (hpf) to 17α-ethinyloestradiol (EE_2_). These embryos showed specific mosaic expression of the reporter. The injected embryos exposed to 1000, 100 and 10 ng/L of EE_2_ expressed GFP in cells most strongly in the somite muscle, heart and liver (72 hpf). This experiment was run in duplicate and was repeated at least seven times.

At 72 and 96 hpf there was no GFP expression detected in embryos in the unexposed controls (Figure 2, Ai-Aiii, Additional file 1: Figure S1), but GFP expressing cells were seen clearly after exposure to EE_2_ (Figure 2, Ci–Diii, Additional file 1: Figure Bi-Bii). No specific GFP expression was observed in the liver at the lowest exposure concentration of EE_2_ (10 ng/L), but weak GFP expression signals were detected in the heart and somite muscle for this exposure concentration (Figure 2, Bi-Biii). GFP expression was most clearly seen in the higher EE_2_ exposures. For exposure to 100 ng EE_2_/L GFP was detected in a feint and dispersed manner in the liver and there was strong GFP expression in the heart and somite muscle. GFP expression in the heart was confirmed by the periodical contractile movement of the GFP expressing cells with each heart beat in the live injected larvae. Strong GFP expression was also observed in the anterior somite muscles. For exposure to 1000 ng EE_2_/L, GFP expression was induced strongly in the liver, somite muscle and heart (Figure 2, Ci-Ciii). Moreover, many GFP expressing cells were observed in the anterior lateral part of the injected embryos (Figure 2, Di). The number of cells expressing GFP appeared to be positively associated with the concentration of the EE_2_ exposure (Table 1). For the transgenic lines of zebrafish that have now been developed, it has bee shown that oestrogenic EDCs activate ERE in liver [34,42], forebrain [41], neuromast [49], heart [36,49] and somite muscle [49] and this is consistent with the expression patterns seen here our transient assay.

**Table 1:** Percentage of plasmid injected zebrafish expressing GFP in different body tissues after exposure to oestrogens (at 72 hpf)

	**Control**	**EE**_**2**_** (ng/L)**			**E**_**2**_**(ng/L)**		**NP (μg/L)**
		**1000**	**100**	**10**	**1000**	**100**	**10**
Liver	0	74.3% (223/300)	61.6% (185/300)	0	70.7% (212/300)	18.7% (56/300)	0
Heart	0	82.7% (248/300)	66.3% (199/300)	0	78% (234/300)	65% (195/300)	56% (168/300)
Muscle	13.3% (40/300)	90.3% (271/300)	78.3% (235/300)	27.3% (82/300)	87% (261/300)	74% (222/300)	71.3% (214/300)
Forebrain		33% (99/300)	0	0	25.6% (77/300)	0	0
Otic vesicle	19% (47/300)	26.3% (78/300)	16.6% (50/300)	10.6% (32/300)	27% (81/300)	25.3% (76/300)	0
lateral line neuromast	10% (32/300)	22.3% (67/300)	13% (39/300)	14.7% (44/300)	25.3% (76/300)	18% (54/300)	4% (12/300)

**Figure 3:**
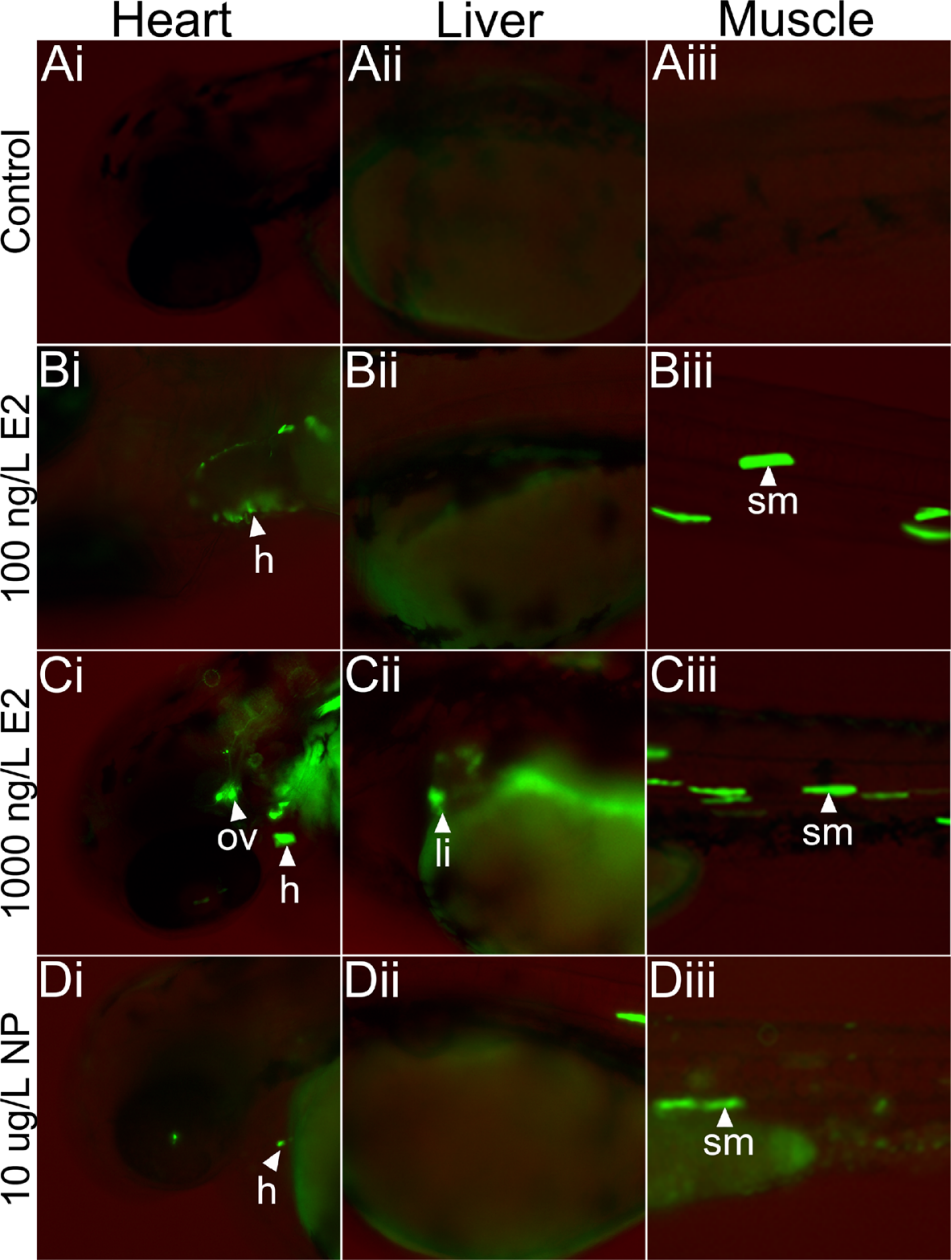
**Comparison of detectable response concentrations for different oestrogen compounds in the zebrafish embryo transient assay.** Injected embryos were exposed for up to 72 h post-fertilisation to 17β-oestradiol (E_2_) and nonylphenol (NP) and fluorescence detected using fluorescence microscopy (Leica DMI 4000 B). No GFP expression was observed in controls (A1-A3), but low level GFP expression occurred in the liver for exposure to 1000 ng E_2_/L (C2) and high level GFP expression was detected in the somite muscle and heart at exposure concentrations of 100 and 1000 ng E_2_/L. Weak GFP expression was also detected in the heart and somite muscle of embryos for exposure to 10 μg/L NP (D1-D3). Embryos exposed to the higher concentrations of NP (100 μg/L) died. This experiment was run in duplicate and was repeated at least seven times.

Exposure to the natural steroid oestrogen, E_2_, resulted in similar responses to that seen for EE_2_. No GFP expression was observed in unexposed controls (Figure 3, Ai-Aiii). Similarly, no GFP expression was observed for the lowest E_2_ exposure concentration (10 ng E_2_/L, data not shown). No GFP expression was observed in the liver at 100 ng E_2_/L, but it was seen in the muscle (somite and cranial) and heart (Figure 3, Bi-Biii). Exposure to 1000 ng E_2_/L strongly induced GFP expression in the heart, otic vesicle and somite muscle and there were some GFP expressing cells observed in the liver (Figure 3, Ci-Ciii). With E_2_, as well as with EE_2_, the GFP signal was also observed in the lateral line neuromast cells albeit a lower frequency (Additional file 1: Figure S1, Table 1). Exposure to 10 μg NP/L induced low level GFP expression in the heart and somite muscle (Figure 3, Di-Diii). The reason for weak GFP induction by NP might be due to a lower sensitivity of early development stages to this chemical in the zebrafish, as has been suggested previously [50]. In addition, it has been reported that NP might be transformed in fish to less potent metabolites [51,52]. These data indicate that different xeno-oestrogens have different target tissue responsiveness and therefore potentially different health effects.

To assess the consistency of transient expression of GFP, we counted the number of GFP-positive embryos for different tissues at 72 hpf. As shown in Table 1, for controls no GFP expression was observed in the liver, heart and forebrain, but weak GFP expression signals were detected in some embryos in somite muscles (13.3%) and the otic vesicle (16%). In contrast, in injected embryos (72 hpf), strong GFP expression occurred in the muscle and heart on exposure to both EE_2_ and E_2_. This was also the case for the liver in injected embryos exposed to both EE_2_ and E_2_: 74% for 1000 ng EE_2_/L, 62% for 100 ng EE_2_/L and 71% for 1000 ng E_2_/L. For the forebrain, the proportion of embryos expressing GFP were 33% and 26% for exposure to 1000 ng EE_2_/L and 1000 ng E_2_/L, respectively. For the NP exposure, the proportion of injected embryos showing GFP expression in muscle was 71% and 56% for the heart.

**Figure 4:**
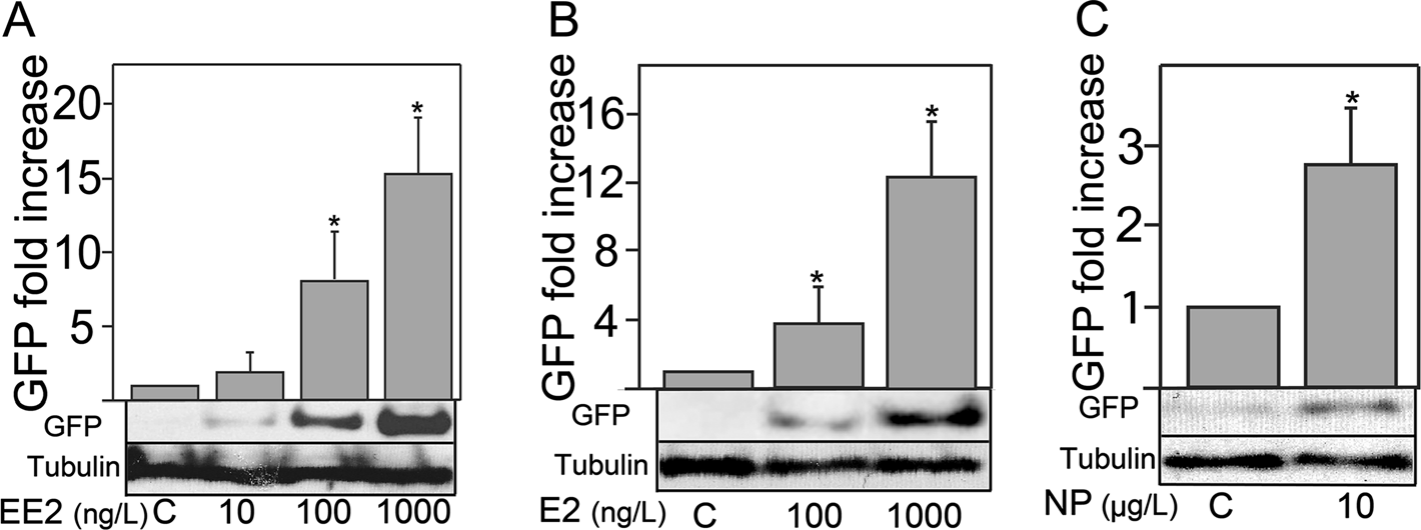
**Western blot analysis of 72 hour old embryos injected with the mixture of the plasmid pERE-Gal4ff containing the*Tol2*transposase mRNA and the plasmid pUAS-GFP.** Whole-body homogenate samples of 40 embryos exposed to 17β-oestradiol (E_2_), 17α-ethinyloestradiol (EE_2_) and nonylphonol (NP) at various exposure concentrations (via the water) are shown. Western blot analysis showed no detectable expression of GFP (Green fluorescent protein) in controls. The level of GFP expression was induced in a concentration dependent manner for the various oestrogenic chemicals (EE_2_, E_2_ and NP). GFP expression level was quantified by using Image J (http://rsb.info.nih.gov/ij/index/html). Error bars represent the standard deviation. This experiment was run in duplicate three times.

In general, the expression of GFP appeared to show a concentration-dependent response for exposure to the test oestrogens, as quantified via western blotting. The highest EE_2_ exposure group (1000 ng/L) indicated a 16.6-fold increase in GFP levels in whole embryos compared with untreated controls. Exposure to 10 ng EE_2_/L resulted in a 2.1-fold increase in GFP compared with the control (Figure 4A). Exposure to E_2_ at 1000 ng/L induced a 12.4-fold increase in GFP above controls and a 2.8-fold increase above controls for exposure to10 μg NP/L (Figure 4B-C). For the highest exposures to EE_2_ and E_2_ (1000 ng/L), the level of GFP induction for EE_2_ was stronger than for E_2_, indicating that EE_2_ was more potent than E_2_.

Assessments of the relative potencies of various environmental oestrogens in our transient assay were consistent with those reported in the literature for other assays systems, with EE_2_ being the most potent compound compared with E_2_, NP and BPA. Legler et al. (2002) [33] similarly reported that EE_2_ was the most potent (xeno) oestrogen compared with E_2_, NP and di(2-ethylhexyl)phthalate (DEHP) in their transgenic zebrafish assay. Relative potency estimates for these chemicals however, can vary depending on the assessment method. For example EE_2_ was shown to be 1.25-fold more potent than E_2_ in yeast-based *in vitro* assay [53] but had around a 30 times higher potency than E_2_ and E_1_ for VTG induction (measured as a protein in the plasma or VTG mRNA in the liver) in female zebrafish [54] in *in vivo*. Thorpe et al. (2003) [55] using a VTG induction assay *in vivo*, reported that EE_2_ was between 11 to 27 times more potent than E_2_. The responses to oestrogen in our zebrafish transient assay developed have shown it to be as sensitive as some other widely used oestrogen detection systems in fish (e.g. VTG induction), and as sensitive as available oestrogen responsive TG fish [35].

### Induction of GFP in the medaka embryo transient expression assay

**Figure 5:**
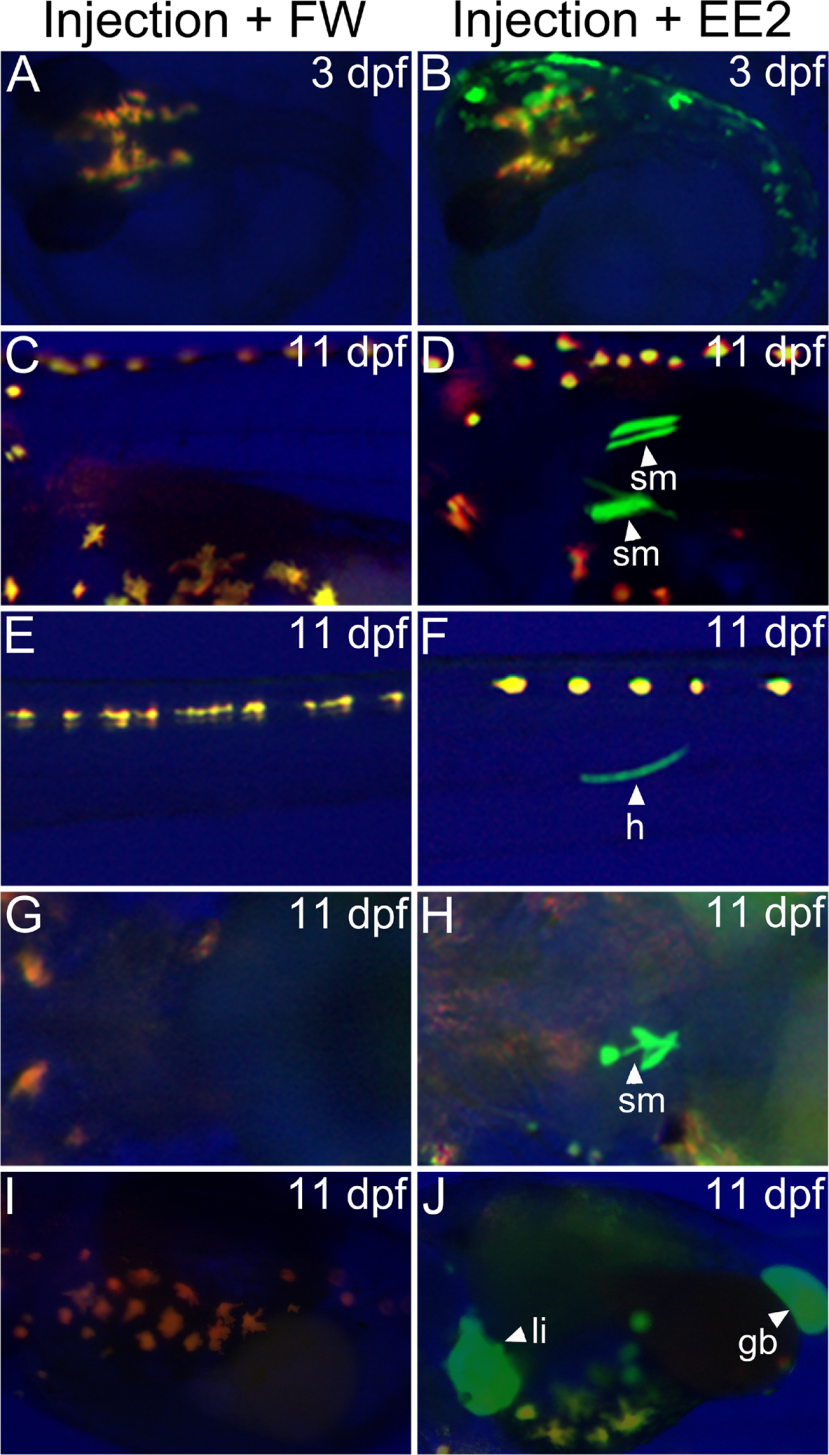
**Analysis of transient expression of GFP in response to oestrogens in medaka embryos.** The plasmid pERE-Gal4ff containing the *Tol2* transposase mRNA and the plasmid pUAS-GFP were injected into 1–2 cell stage medaka embryos and the embryos were exposed to 17α-ethynyloestradiol (100 ng EE_2_/L) via the water. No GFP expression was detected in controls. The injected medaka embryos exposed to EE_2_ showed strong GFP expression in the skin in the anterior region and in the body trunk (B). At 11 dpf, fish exposed to EE_2_ showed GFP expression in the somite muscle, heart, liver and gall bladder (D, F, H and J).

To examine if our transient assay system was applicable in other fish species, we assessed the response of GFP in the medaka embryo (Figure 5). Medaka has four types of pigment cells, namely melanophores, xanthophores, leucophores and iridophores [56] that could interfere with the detection of GFP. To avoid this, we imaged the embryos using normal light, green (GFP) and red (RFP). This was done because pigment cells are auto-fluorescent both in green and red spectra, and therefore by overlaying the different images (blue as background) we were able to distinguish the signal due to GFP. At 3 dpf, injected medaka embryos showed GFP expression in the skin, whereas no GFP expression was detected in the control (unexposed embryos). To enhance our ability to see clear GFP responses in other tissues, we exposed the injected embryos for 11 days. At this time, tissue specific expression became obvious in the liver, heart, gall bladder and somite (stage 40; [57]). Medaka showed a lower level of expression of GFP in muscle compared with zebrafish (See Figure. 2Ciii and Figure 5D, F) which might be due to differences in the transgenesis efficiency with *Tol2* system between the two species (being lower in medaka than in zebrafish [58]). However, the different responses between the species may also be due to the physiological and genetic differences between them.

GFP expression observed in the skin and gallbladder in medaka was not observed in zebrafish, which might suggest that oestrogen receptor expression varies between fish species or oestrogen receptor binding affinities for different fish species might be different in the different embryos, at least for these organs. Differences between fish species have been shown for tissue distribution of ER subtypes as well as differences in ER affinities for environmental oestrogens ([59-61]). We do not yet know how the oestrogenic response pattern observed by GFP expression is related to the expression of ERs. The available literature suggests that ERs are expressed ubiquitously in the whole embryos both in zebrafish and medaka with an elevated expression in the neuromast cells in zebrafish [16,62]. Given this, the tissue specific response of GFP to oestrogenes may be as a consequence of the co-factors associated with ER signalling pathway.

## Conclusion

Despite the fact that transient expression analysis has been used in various areas of developmental biology, there has been no report using transient expression analysis for detection of oestrogen compounds. The vector system we have developed and applied in a transient assay effectively detects oestrogenic responses in body tissues. Furthermore, the transient assay system is as sensitive as many of the available TG fish for detecting oestrogens, in some cases considerably more so [63] There are however, exceptions to this, where the most sensitive TG fish developed have been shown to respond to oestrogens at 1 ng EE_2_/L (5 ng E_2_/L) [49] and 10 ng EE_2_/L (300 pM E_2_ (approximately 81 ng E_2_/L)) [33,35]. Drawbacks of the transient expression assay over TG fish includes the fact that mosaicism can make it more difficult to detect GFP expression in physically small tissues such as lateral line neuromast.

Comparing our transient assay system with VTG induction, a well established biomarker of oestrogen exposure, the detection threshold for EE_2_ (as an example) in zebrafish *in vivo* is 2 ng EE_2_/L [64]. Induction of VTG in medaka and common carp hepatocytes in culture is reported at 30 ng E_2_/L [65] and 0.01 μM E_2_[66], respectively. The EC50 concentration (half maximal effective concentration) for EE_2_ in the MCF-7 cell assay and yeast-oestrogen screen (YES) from Folmar et al. (2002) [67] is 0.017 nM (5 ng/L) and 0.29 nM (86 ng/L), respectively.

The vector system developed detected oestrogenic EDCs in both the zebrafish and medaka, indicating the likely wide suitability for application to other fish species. This system would be a particularly powerful technique for use in fish species with long generation times and/or where there are other difficulties for generating transgenic lines in those species. The transient expression assay further provides a novel *in vivo* system for investigating oestrogenic effects on embryo development in fish.

## Methods

### DNA construct for developing a transient assay

The construct, called pTol2_3ERE-TATA-Gal4ff was based on the sequence of EREc38 (EREc38 5’-CCAGGTCAGAGTGACCTGAGCTAAAATAACACATTCAG-3’) published by Sathya et al. (1997) [39]. The construct was generated by Polymerase Chain Reaction (PCR) using primers (upstream primer CCAGGTCAGAGTGACCTGAGCTAAAATAACACATTCA GCC AGGTCAGAGTG and downstream primer CTGAATGTGTTATTTTAGCTCAGG TCACTC TGACCTGGCTGAATGTGTTAT). The following conditions were used for 8 PCR cycles : denaturation at 96 °C for 1 min, annealing at 60 °C and extension at 72 °C for 1 min. Multiple tandem copies of EREc38 (nEREc38, where n = number of ERE copies) were obtained by PCR. EREc38 oligomers were prepared by restriction digestion of the 3ERE with *SmaI.* The reporter gene vector pKS(+) was digested with *XhoI* and *XbaI*. After obtaining the 3ERE fragment, a second fragment called TATA was constructed following the same protocol, using the following primers: (upstream primer GGCGTCGACTCTAGAGGGTATATAATAGAT CTGCGATCTAAGTAAGCTTGG downstream primer CGCGGGCCCGGCTTTACCAACAG TACCGGAATGCCAAGCTTACTTAGATCG). TATA was digested with *ApaI* and *SalI*. Gal4-ff (amplified by PCR using primers: upstream primer GCCGGGCCCGCCACCATGAAGCTA CTGCTGTC TTCT downstream primer CGCGGTACCGATTAGTTACCCGGGAGC) was inserted into the vector which already contained the 3ERE and TATA. The *BamHI* and *NotI* fragments of p3ERE-TATA-Gal4ffwere inserted into the *BamHI* and *NotI* sites of the plasmid pBR322 vector [30], the latter containing the *Tol2* transposon. The positive clones were confirmed by sequencing. The resulting plasmid was designated as pTol2_3ERE-TATA-Gal4ff.

### Fish maintenance and microinjection of embryos with DNA

Zebrafish were maintained at 28 ± 1 °C under a 14 h light/10 h dark photoperiod and were fed Tetramin dry tropical flake food (Tetramin; Tetraweke, Melle, Germany) twice a day. Fertilised zebrafish eggs were collected within 20 minutes post-fertilisation. Medaka were maintained at 28 °C on a 14:8 light:dark cycle. All adult medaka in this study were fed *Artemia* twice a day. The concentration of plasmid was 20 pg/nl and 0.05% phenol red was added to make the solution visible. Under a dissecting microscope, fertilised eggs were placed in injection plate and eggs were microinjected with a syringe into the centre of the cell over the yolk-cytoplasm boundary. Embryos were microinjected at the 1–2 cell stage with 2 nl DNA using a microinjector (INTRACEL, PICOSPRITZER®III). Some of the embryos were also exposed to various chemicals as detailed below.

### Chemicals

17α-ethinyloestradiol (≥98% purity). 17β-oestradiol (98% purity), and 4-Nonylphenol (Acros Organics) were purchase from Sigma Chemical Co. Ltd. The stock solutions of EE_2_, E_2_ and NP were prepared in acetone and subsequently stored at 4 °C, until required for the exposures. The working solutions were prepared 3 days before use in the exposures. Prior to exposure, the solvent was evaporated under a stream of nitrogen and the working solutions made up with water and stirred vigorously for 1 day. The nominal concentration of treatment solutions were: 17α-ethinyloestradiol (EE_2_): 1000 ng/L, 100 ng/L and 10 ng/L; 17β-oestradiol (E_2_): 1000 ng/L, 100 ng/L and 10 ng/L; 4-Nonylphenol (NP): 100 μg/L and 10 μg/L Chemicals were dissolved in embryo culture water. Fifty embryos per chemical concentration were exposed for up to 72 hours at 28 °C. All experiments were run in duplicate and were repeated at least seven times.

### The transient expression assay using fluorescent microscopy detection

The plasmid pTol2_ERE-TATA-Gal4ff was mixed in a 1:1 ratio with the plasmid pTol2_UAS-GFP and then mixed with transposase mRNA in a ratio of 1:2. The mixture was microinjected (20 pg/nl) into 1–2 cell stage embryos of zebrafish. Injected zebrafish embryos were cultured for up to 72 hours (zebrafish) with and without exposure to E_2_, EE_2_ and NP, and GFP expression examined by fluorescence microscopy (Leica DMI 4000 B). In the studies with medaka, the plasmid pTol2_ERE-TATA-Gal4ff with transposase mRNA was mixed in a 1:1 ratio with the plasmid pTol2_UAS-GFP and this was then injected into 1–2 cell stage medaka embryos (20 pg/nl). Injected medaka eggs were exposed to EE_2_ (100 ng/L) and embryo culture water for 11 days at 28 °C (±1 °C). GFP expression was observed under a fluorescent microscope (NIKON SMZ1500).

### Synthesis of transposase mRNA

Transposase mRNA was synthesised *in vitro* using pCS-TP which carries *Tol2* transposase cDNA. The plasmid pCS-TP was digested with *NotI* and purified by phenol: chloroform extraction and ethanol precipitation. RNA synthesis was performed using mMessage mMachine SP6 kit (Ambion). The synthesized mRNA was purified with Quick spin column for radiolabeled RNA purification (Roche), purified by phenol: chloroform extraction and by the subsequently ethanol precipitated. The RNA precipitated was suspended in distilled water and used for co-microinjection with pTol2_ERE-TATA-Gal4ff.

### Western blot analysis

Western blot analysis was employed to quantify GFP expression. Embryos were transferred to a 1.5 ml tube filled with lysis buffer (2x LDS (Lithium dodecyl sulfate) sample buffer (Invitrogen), 2-Mercapto ethanol and distilled water), incubated at 95 °C for 5 minutes and the vessel tapped to get them to the bottom of tube. Samples were homogenised five times and centrifuged for 1 min at 1000 rpm. Protein samples were applied to 5% polyacrylamide-SDS gel and subjected to electrophoresis at 110 V for 2 hour and separated proteins were transferred to nitrocellulose membrane. The membranes were blocked for 1 hour in blocking solution (5% skimmed milk in 1x phosphate buffered saline (PBS) + 0.1% Tween (PBSTx)), and washed three times with distilled water (ddH_2_O). The membranes were incubated overnight at 4 °C with primary antibody, rabbit anti-GFP (ams Biotechonology), diluted 1:2500 in blocking solution. The membranes were washed for 3 x 15 minutes in PBSTx. The membranes were incubated with HRP-Goat Anti-Rabbit IgG (Invitrogen, Carlsbad, U.S.A) at 1:2000 in blocking solution for 2 hours and then washed 3 x 15 minutes in PBSTx again. For detection, Western blotting luminol reagent (Thermo Scientific) was used. The intensity of GFP was analysed using Image J (http://rsbweb.nih.gov/ij/), normalised to the intensity of alpha-tubulin band and indicated as fold increase in GFP over the level in control larvae.

### Statistical analysis

All data are reported as mean ± SEM. Statistical evaluation were performed using Student’s *t*-test and the comparison between controls and each exposed groups. Statistical significance is indicated at the p <0.05 level.

## Competing interests

The authors declare that they have no competing interests.

## Authors’ contributions

All authors designed the study. OHL performed all the experiments and analysed the data. The manuscript was written by all authors (OHL, TK and CRT). All authors read and approved the final manuscript

## Supplementary Material

Additional file 1 **Figure S1 GFP expression at 4 dpf embryos in the transient expression assay.** Injected embryos without chemical exposure (A) or exposed to the oestrogenic chemicals 17α-ethinyloestradiol (1000 ngEE_2_/L) (B), 17β-oestradiol (1000 ng E_2_/L) (C) and nonylphonol (10 μgNP/L) (D) for 4 days. Head with lateral (L) and ventral (V) views (i and ii) and trunk with lateral view (iii). The shape of the liver is outlined with a white line. No GFP expression was observed in the unexposed control (A). EE_2_ and E_2_ induced GFP expression in the heart (h), liver (li), neuromasts (n) and somite muscles (sm) (B,C). In NP exposed larvae, GFP expression was observed mainly in the muscle and heart (D). Click here for file
