# Supplementary figures and images for: Development of a transient expression assay for detecting environmental oestrogens in zebrafish and medaka embryos
Source: BMC Biotechnol. 2012 Jun 24;12:32. doi: 10.1186/1472-6750-12-32 (PMC3410757; doi:10.1186/1472-6750-12-32)

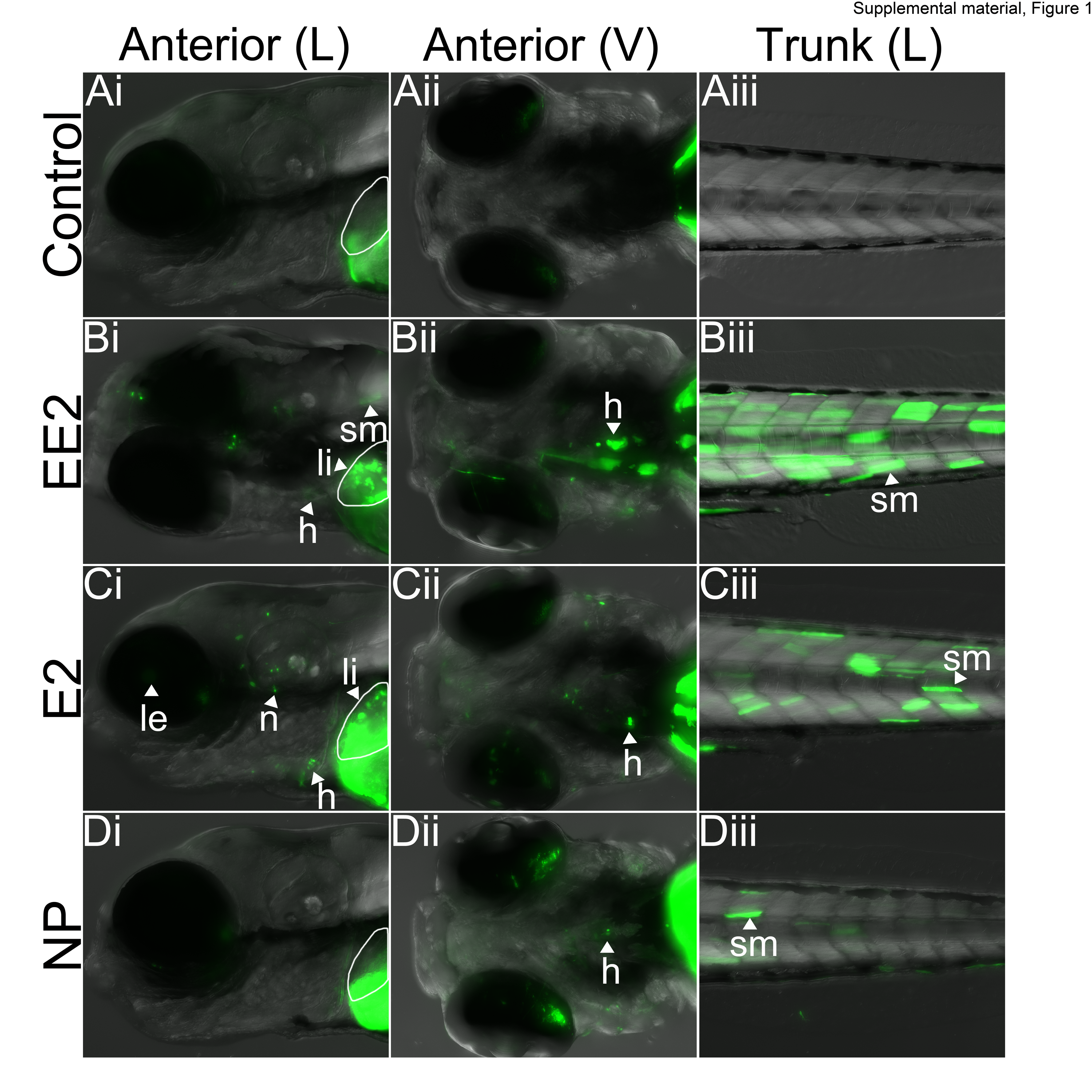

Supplement: Additional file 1 — Figure S1 GFP expression at 4 dpf embryos in the transient expression assay. Injected embryos without chemical exposure (A) or exposed to the oestrogenic chemicals 17α-ethinyloestradiol (1000 ngEE2/L) (B), 17β-oestradiol (1000 ng E2/L) (C) and nonylphonol (10 μgNP/L) (D) for 4 days. Head with lateral (L) and ventral (V) views (i and ii) and trunk with lateral view (iii). The shape of the liver is outlined with a white line. No GFP expression was observed in the unexposed control (A). EE2 and E2 induced GFP expression in the heart (h), liver (li), neuromasts (n) and somite muscles (sm) (B,C). In NP exposed larvae, GFP expression was observed mainly in the muscle and heart (D). [file 1472-6750-12-32-S1.tiff]
